# Multi-Task Deep Supervision on Attention R2U-Net for Brain Tumor Segmentation

**DOI:** 10.3389/fonc.2021.704850

**Published:** 2021-09-17

**Authors:** Shiqiang Ma, Jijun Tang, Fei Guo

**Affiliations:** ^1^School of Computer Science and Technology, College of Intelligence and Computing, Tianjin University, Tianjin, China; ^2^Shenzhen Institute of Advanced Technology, Chinese Academy of Sciences, Shenzhen, China; ^3^Department of Computer Science and Engineering, University of South Carolina, Columbia, SC, United States; ^4^School of Computer Science and Engineering, Central South University, Changsha, China

**Keywords:** brain tumor segmentation, attention mechanism, multi-task learning, semi-supervised learning, multi-scale feature fusion, deep supervision

## Abstract

Accurate automatic medical image segmentation technology plays an important role for the diagnosis and treatment of brain tumor. However, simple deep learning models are difficult to locate the tumor area and obtain accurate segmentation boundaries. In order to solve the problems above, we propose a 2D end-to-end model of attention R2U-Net with multi-task deep supervision (MTDS). MTDS can extract rich semantic information from images, obtain accurate segmentation boundaries, and prevent overfitting problems in deep learning. Furthermore, we propose the attention pre-activation residual module (APR), which is an attention mechanism based on multi-scale fusion methods. APR is suitable for a deep learning model to help the network locate the tumor area accurately. Finally, we evaluate our proposed model on the public BraTS 2020 validation dataset which consists of 125 cases, and got a competitive brain tumor segmentation result. Compared with the state-of-the-art brain tumor segmentation methods, our method has the characteristics of a small parameter and low computational cost.

## 1. Introduction

Brain tumors are the most common primary malignant tumors of the brain caused by the canceration of glial cells in the brain and spinal cord. Brain tumors have the characteristics of high morbidity and mortality. Automatic segmentation technology of brain tumor can assist professional doctors to diagnose brain lesions and provide imaging technical support for the diagnosis and treatment of brain tumor patients. With the development of convolutional neural networks, the brain tumor automatic segmentation technology based on deep learning had achieved a high segmentation accuracy. However, the location of brain tumor regions and accurate segmentation of tumor edges have always been the difficulties of deep learning methods. In order to obtain accurate segmentation results, deep learning methods usually require a numerous parameters and a long calculation time, which leads to extremely high demands on the hardware. Therefore, it is of great significance to develop a simple and efficient network architecture.

Since 2015, a variety of Convolutional Neural Networks (CNN) architectures for brain tumor segmentation have been proposed. Havaei et al. proposed the InputCascadeCNN model (1), which used cascaded CNN to segment brain tumor regions. After the network obtained a small feature map, it used two CNN branches with different convolution kernel sizes to further extract local feature and global information, and fused multi-scale information. Dvorak et al. proposed a 6-layer CNN, the brain image was cropped into multiple patches, and these patches were clustered using k-means to obtain N clustering results and formed a dictionary as the input of network (2). Pereira et al. used a 3X3 convolution kernel to extract the segmentation features (3), like VGG (4). When the receptive field of the same size was obtained, a smaller convolution kernel could effectively reduce the amount of network parameters and enabled the network to be designed deeper. At the same time, the author used intensity normalization in the data preprocessing process. Kamnitsas et al. proposed DeepMedic (5), using residual block (6) in the CNN architecture. DeepMedic used images of different resolutions as the input of two branch networks to obtain multi-scale information and fused the multi-scale information. Randhawa et al. (7) used a classification network to classify each input pixel. Kamnitsas et al. proposed EMMA (8), which merged the outputs of multiple independent networks through an average confidence.

Although a variety of network structures have been proposed, the location of tumor regions and accurate segmentation of tumor boundaries have always been the difficulties of brain tumor segmentation. The traditional deep learning method usually used the fully connected layer as the last layer of the network, but one-dimensional probability information will lose the spatial structure information of the image, which is not suitable for image segmentation. Fully convolutional neural networks (FCN) (9) and U-Net (10) used a fully convolutional layer as the last layer of network, and used an up-sampling operation that is symmetrical to down-sampling to keep the size of the feature map consistent with the input size of the network. This method effectively improves the ability of neural network to locate the region of interest (ROI). However, the shape and pixel intensity of brain tumor data are affected by differences between patients and data collection agencies, which makes it difficult for traditional U-Net and FCN to obtain accurate location and segmentation accuracy when the number of parameters is small.

In order to further improve the performance of the U-Net architecture, a variety of improved U-Net architectures have been proposed. DCSNN (11) extends the architecture of U-Net with a residual module by adding a symmetric mask in multiple layers. Isensee et al. proposed an improved U-Net architecture (12), which used the pre-activation residual block (13) as the basic unit of network. At the same time, the leaky rectified linear unit (leaky ReLU) was used to prevent the gradient from disappearing, and batch normalization (14) was replaced with instance normalization (15), which improved the stability of the network for a feature extraction of small batches. nnU-Net (16) used 2D U-Net, 3D U-Net, and cascaded 3D U-Net to adaptively segment inputs of different resolutions. Although most of the improved u-net methods improve the segmentation accuracy, they also increase the depth, parameters, and computing time of deep learning network.

The depth of the network and the size of the parameters will directly affect the ability of feature extraction, usually a deeper network structure and larger parameters will improve the segmentation accuracy. However, the increase of parameters will lead to an over fitting problem and reduce the robustness of the network. Too deep network structure will lead to the problem of vanishing gradient and exploding gradient in network training. In order to solve the vanishing gradient problem and exploding gradient problem of the deep network, deep supervision methods were introduced (17–19). In theory, when the size of convolution kernel remains the same, as the number of network layers becomes deeper, the network gained a stronger nonlinear expression capability. However, with the deepening of the network, backpropagation becomes difficult, resulting in a decrease in network performance. Chen et al. proposed VoxResNet, which was used in brain segmentation. In order to solve the problem of automatic segmentation caused by the difference in the shape of 3D image slices, the author merged the deep supervision results containing multi-level context information as the final output of network (20). Zeng et al. used a multi-level deep supervision of 3D U-Net to alleviate the potential gradient vanishing problem in a Proximal femur segmentation (21). Zhang et al. used deep supervision in a retinal vessel segmentation to learn a better semantically representation and help convergence (22). Zeng et al. proposed a multi-scale deep supervision method in infant brain MR image segmentation, which addresses that the final loss cannot supervise a shallow fracture extraction (23).

Similarly, a deep supervision method was also used in the brain tumor segmentation (12). Deep supervision usually used the same label to perform a single task, mainly focusing on solving the problem of gradient vanishing. When Resnet was proposed, the problem of gradient vanishing was effectively improved. Andriy Myronenko proposed a multi-task learning method (24), which used U-Net to perform brain tumor segmentation tasks and used another decoder branch for image reconstruction. This method was similar to a deep supervision, replacing the label of a decoder branch with a reconstruction label, thereby preventing the problem of network overfitting. Similarly, Chen et al. proposed the Multi-task Attention-based Semi-Supervised Learning (MASSL) framework, which used soft segmentation to obtain pseudo-labels of tumor and non-tumor regions, and used pseudo-labels to supervise the reconstruction branch (25). They proposed that multi-task learning could improve the capture of segmentation features in the encoder part. Jiang et al. used two decoder branches with different up-sampling structures to help the encoder part to collect more abundant brain tumor regional features (26). Weninger et al. used the three tasks of segmentation, classification, and reconstruction to jointly train the shared encoder part (27). The methods above used other related tasks as labels for deep supervision, and obtained accurate brain tumor segmentation results. It showed that the deep supervision method could not only improve the vanishing gradient problem of deep network, but also enabled the network to learn a richer visual representation and prevented overfitting.

In the brain MRI image of the patient, the brain tumor area is small, so the brain tumor segmentation has a problem of class imbalance. In order to focus on the brain tumor area, the visual attention mechanism was introduced into the medical image segmentation network. Hu et al. used the global max-pooling layer to adaptively calculate the weight of each channel, and feed the weight back to the feature channel (28). On this basis, Li et al. designed a dynamic selection mechanism for the convolution kernel based on the working principle of visual neuron, and adaptively adjusted the receptive field size obtained by the convolution kernel through multi-scale information, and used softmax to Features of different sizes are merged (29). Woo et al. used the channel attention module and spatial attention module to adaptively select the beneficial channel features and spatial features, and used element-wise summation and sigmoid activation function to fuse the two features (30).

In this paper, we proposed a new end-to-end brain tumor segmentation network. We made partial modifications to the Attention U-Net (31) framework and design MTDS and APR module. Our work aims to enhance the ability of network to capture the features of brain tumor and reduce the impact of class imbalance, and improve the accuracy of brain tumor segmentation.

## 2. Methods

The detailed description of our proposed automatic brain tumor segmentation method will be given in this section. The proposed deep learning model architecture is presented, including the UNet-like basic network, APR module, and MTDS.

### 2.1 Basic Network

The design of the model needs to consider the distribution characteristics of the dataset. Compared with natural images, medical images are symmetrical and have a simpler semantic information and a more fixed image structure. However, medical images often contain noise and artifacts, and the boundary information is blurred. In the view of a single structure and the fuzzy boundary of medical images, the autoencoder structure with skip connection has become the benchmark for brain tumor segmentation. The structure of convolutional autoencoder can reduce the amount of network parameters while obtaining high-level semantic features, saving computing resources. Skip connection combines low-level and high-level features to help the network reconstruct the detailed information of ROI. Our basic network is similar to Attention U-Net. In order to obtain a higher tumor segmentation accuracy, we adjusted the structure of the network.

**Figure 1:**
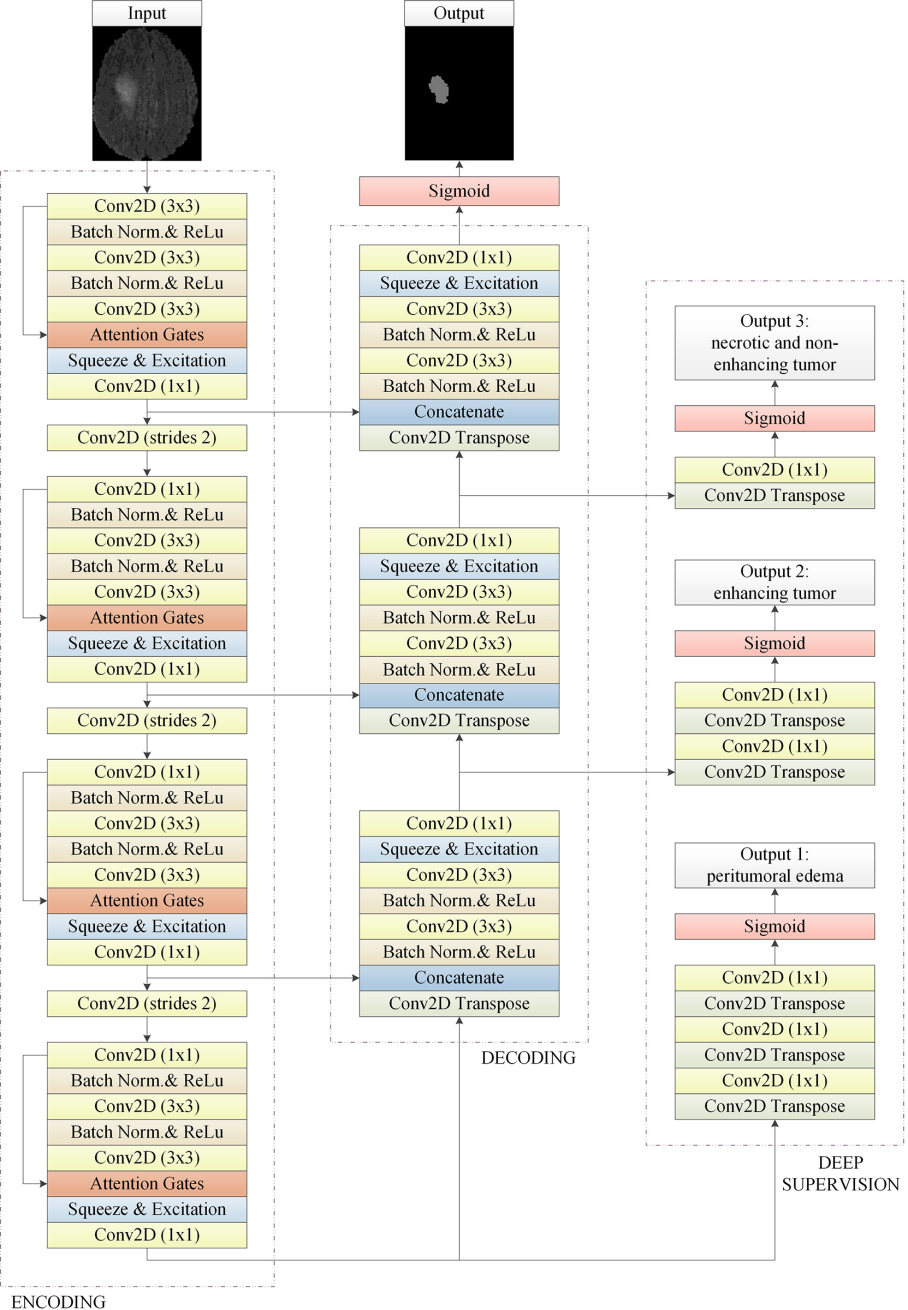
The basic 2D convolutional neural network for brain tumor segmentation. It consists of encoding, decoding, and deep supervision. Our approach is an end-to-end network, the input of the network is a 2D image composed of four modes, and the output is the whole brain tumor prediction result of each 2D image. Output1, output2, and output3 are the subregions of the brain tumors, which are the peritumoral edema, enhancing tumor, and the necrotic and the non-enhancing tumor, respectively. Multi-task deep supervision with progressive relationships can help our method accurately extract the visual features of each stage.

**Figure 2:**
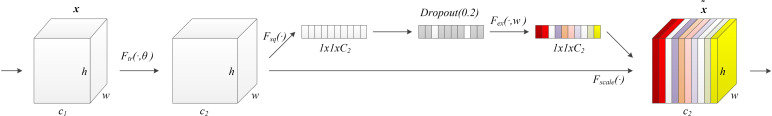
Our proposed SE module with the dropout layer. Adding the dropout layer can prevent overfitting and improve the robustness of the deep learning network. The SE module assigns different weights to the feature channels to help the network obtain the most effective features of the brain tumor regions.

The model structure is shown in **Figure 1**, similar to LinkNet (32), we combined the U-Net structure and the ResNet structure. According to the statement in (33), the skip connection of U-Net cannot eliminate the vanishing gradient problem, but the shortcut of ResNet can prevent the vanishing gradient problem. In addition, the skip connection of U-Net helps to increase the convergence speed the same as the shortcut of ResNet. The main structure includes encoder, decoder, and deep supervision. Encoder consists of 3 down-sampling, 4 APR module, and 4 Squeeze-and-excitation (SE) modules. For the first Residual Units of the encoder part, the number of convolution kernel is 32, and doubles with each next residual unit. Decoder includes 3 up-sampling, 3 pre-activation convolution blocks, 3 SE modules, 1 convolutional layer (1x1), and 1 sigmoid. In the SE module, some channels are considered to have no important contribution to the segmentation task, and their weights are very small, which leads to overfitting and vanishing gradients problem. Therefore, we added the dropout layer to prevent the network from overfitting and improve the robustness of the deep learning network. The random change of channel weight helps the network learn the visual expression of different channel features in brain tumor segmentation. The experimental results also prove this conclusion. The SE module is shown in **Figure 2**, and **Table 1** reports the results of comparative experiments with or without dropout in the SE module.

**Table 1:** The results of comparative experiments with or without dropout in the SE module on the BraTS 2020.

Method	DSC (%)	Sensitivity (%)	Specificity (%)	Hausdroff95
without dropout	88.59	88.52	99.86	7.74
with dropout	**89.18**	**89.24**	**99.91**	**5.77**

The bold values indicate the best results.

### 2.2 Multi-Task Deep Supervision

In the brain tumor automatic segmentation model, we use the MTDS method to optimize the training process of deep learning network and extract richer visual features. In the process of back propagation, the deep network converges slowly or even hard to converge due to the problem of vanishing gradient. Deep supervision techniques are used to alleviate the training difficulty of deep networks. However, unreasonable network design affects the hierarchical feature expression ability of the network, and even disrupt the network optimization goal. Usually, the shallow layers of the network extract low-level features in the image, such as boundary information. The deep layers of the network can extract high-level features, in other words, the semantic information of an image. When deep supervision is designed in the front of the network, it forces the network to change the normal learning process, resulting in an inconsistent loss of optimization goals and affecting the segmentation accuracy. This impact became more serious in many deep networks (34).

**Figure 3:**
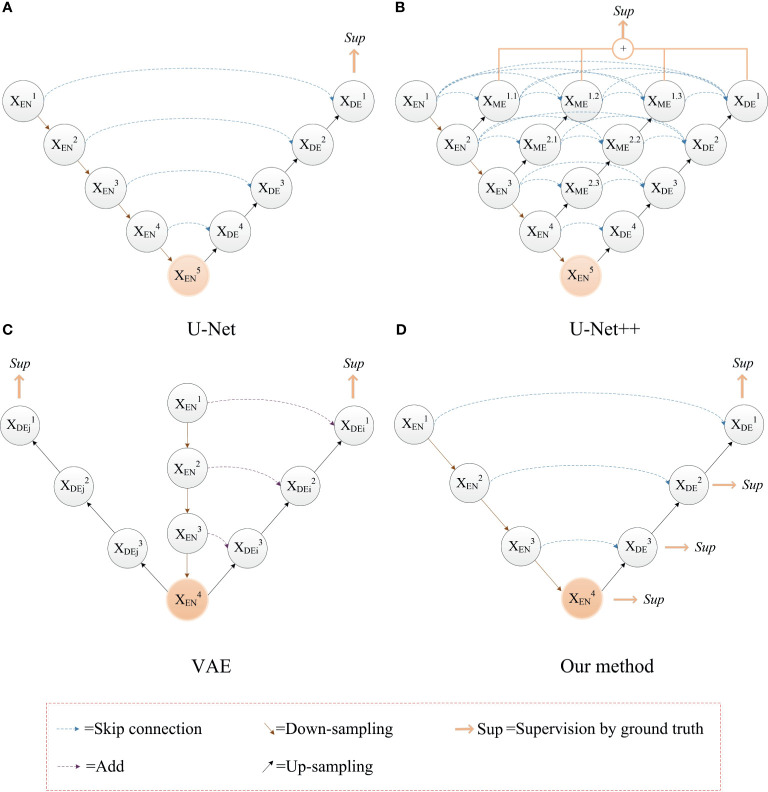
The comparison between our proposed deep supervision method and other methods. **(A)** The U-Net model; **(B)** Use of multiple shortcuts and skip connections: this method adds a deep supervision method to each level of sub network, which affect the hierarchical feature expression ability of network (35); **(C)** Use of image reconstruction task as deep supervision to prevent the network from overfitting (24). **(D)** Our method with deep supervision.

Based on the problems above, we use the ground truth of multiple segmentation tasks as the label for deep supervision, and optimize the training process through multiple associated sub-segmentation tasks. While solving the vanishing gradient problem, the ability of the network to extract segmentation features of a sub-tumor region is improved. The comparison between our proposed deep supervision method and other methods is shown in **Figure 3**. The sub-segmentation task is used as the regularization item of the network to improve the generalization ability of the model and prevent overfitting. Normally, whole tumors consist of the peritumoral edema, enhancing tumor, and the necrotic and the non-enhancing tumor. The area of enhancing tumor is smaller than the area of peritumoral edema and the necrotic and the non-enhancing tumor. High-level semantic information is not conducive to capturing the features of the enhancing tumor area, while low-level boundary information can better express the detailed features of the enhancing tumor. In our method, the enhancing tumor ground truth is used as the label of first deep supervision, and the shallow layers of network can better capture the boundary details of the enhancing tumor area. Segmentation of the necrotic area and segmentation of the peritumoral edema area are respectively used as the other two deep supervision tasks, and the final output of the network is the segmentation of the whole tumor area. The optimization objective of whole brain tumor segmentation and multi-task auxiliary segmentation can be expressed as follows:


(1) $$\arg\underset{\omega_{m},\omega_{\alpha }}{\max}\;L_{m}(\omega_{m};D)+L_{\alpha }(\omega_{\alpha };D)$$

where *D* is the brain tumor datasets with annotation, *ω_m_* is the learnable weight matrices of whole brain tumor segmentation network, and *ω_a_* correspond to the learnable weight matrices of multi-task auxiliary segmentation network. *L_m_* denotes the total loss function of whole brain tumor segmentation, and *L*α is the loss function of multi-task auxiliary segmentation.

### 2.3 Attention Pre-Activation Residual Module

In addition to the function of identity mapping, residual module is a simple multi-scale feature fusion method (36). Multi scale feature representation is very important for image segmentation. Except to the pixel intensity, the morphological features of the tumor region are of great importance for brain tumor segmentation. Learning the difference between the morphological features of brain tumor and the surrounding normal brain tissue by deep convolution network is helpful to the accurate segmentation of the brain tumor region. The combination of the boundary information of the tumor region and its high-level semantic information can make the deep convolution network accurately locate ROI (31). Based on the residual module, the improved multi-scale information fusion of deep convolution network is beneficial to the classification, segmentation, and detection of visual tasks.

**Figure 4:**
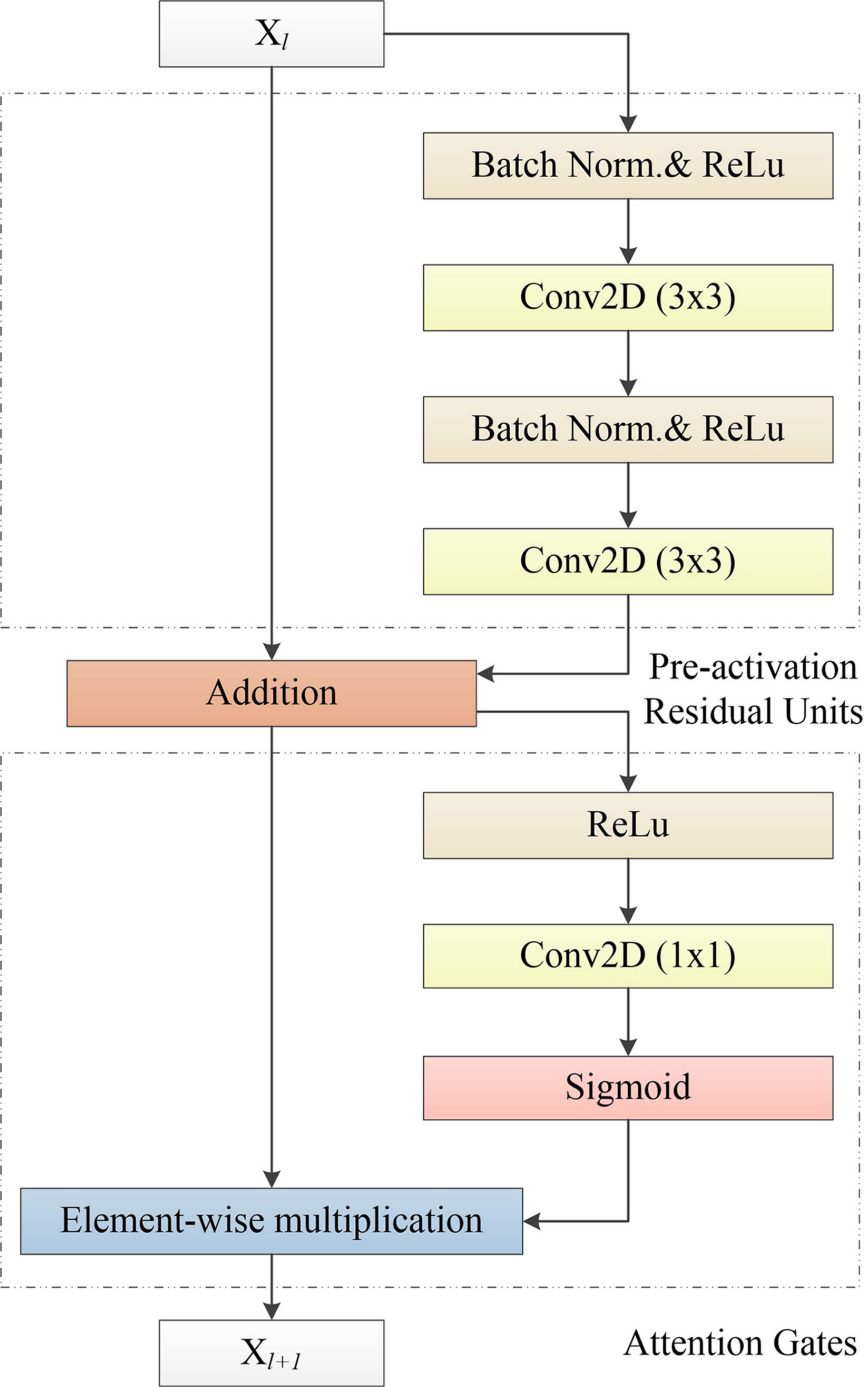
Our proposed APR module, which consists of the Pre-activation Residual Units and Attention Gates. Pre-activation Residual Units obtain feature maps of low-level and high-level scales. Attention Gates obtains the weighted feature map of the 2D image by performing nonlinear processing on the output result of the Pre-activation Residual Units.

Therefore, Res2Net (37) and other network structures are proposed. Res2Net designed a residual structure, which can significantly increase the multi-scale information of the residual module. However, the feature fusion of Res2Net is simple, so that it is difficult to make full use of the multi-scale information. On this basis, we propose an APR module, which is used to improve the attention of the deep network to ROI. This structure combines the pre-activation residual units (13) and attention gates (AGs) (31). The APR module can be seen in **Figure 4**. Thanks to the excellent performance of the pre-activation residual units in the field of medical image segmentation (24, 26, 33, 38), we use the pre-activation residual units as the basic module of the segmentation network. Pre-activation residual units can help information propagation, which include 2 batch normalization, 2 rectified linear unit (ReLU), and 2 weight layers. The output *x_l+1_* of the pre-activation residual units can be expressed as follows:


(2) $$x_{l+1}=x_{l}+F(x_{l},\omega_{l})$$

(3) $$F(x_{l},\omega_{l})=F_{r}(F_{r}(x_{l},\omega_{l}))$$

(4) $$F_{r}(x_{l},\omega_{l})=W_{x}(\sigma_{1}(W_{b}(x_{l},\omega_{l})))$$

where *x_l_* is the input of the pre-activation residual units, *w_l_* is the learnable weight matrices. *F(x_l_,w_1_)* denotes the pre-activation residual function, *F(x_l_,w_1_)* consists of two cascaded subunits *F_r_(x_l_,w_1_)*. An element-wise addition is used to combine the feature map of *x_l_* and *F(x_l_,w_1_)*. Each *F_r_(x_l_,w_1_)* includes a batch normalization *ω_b_*, a ReLU *σ*_1_, and a 3X3 convolutional layer *W_x_*. The 3X3 convolution layer enables the pre-activation residual function to obtain a larger receptive field than the input, which provides multi-scale visual information for the feature fusion of the attention gates.

Attention gates, which is like the shortcut-only gating and 1x1 convolutional shortcut (13), have a stronger visual representational ability. Attention gates consists of a ReLU, 1x1 convolutional layer, and a sigmoid activation function. ROI is selected by analyzing both the activations and contextual information. The output *y_l_*_+1_ of attention gates can be expressed as follows:


(5) $$y_{l+1}=y_{l}⊙F_{\alpha }(y_{l})$$

(6) $$F_{\alpha }(y_{l})=\sigma_{2}(W_{y}(\sigma_{1}(y_{l})))$$

where *y_l_* is the input of attention gates, which is the output of the pre-activation residual units (*y_l_* = *x_l_*_+1_). ⊙ is the element-wise multiplication. *Fα(y_1_)* denotes the attention gates function. *W_y_* is a 1x1 convolutional layer used to compute linear transformation. $\sigma_{2}=\frac{1}{\left(1+exp(-yl)\right)}$ is a sigmoid activation function. ReLU and sigmoid can improve the nonlinear expression ability of the attention gates. In addition, sigmoid can make attention gates parameters have a better convergence.

We combine the pre-activation residual unit and attention gates, and obtain the APR module as follows:


(7) $$y_{l+1}=(x_{l}+F_{r}(F_{r}(x_{l},\omega_{l})))⊙\sigma_{2}(W_{y}(\sigma_{1}(x_{l}+F_{r}(F_{r}(x_{l},\omega_{l})))))$$

APR module is a multi-scale feature fusion method based on the residual unit. This method obtains multi-scale information from the residual units and generates a gating signal to control the importance of features in different spatial regions, to suppress the feature response of irrelevant background regions.

## 3 Experiments and Results

In this section, the brain tumor datasets and the pre-processing methods are introduced. And then, we provide the training details of network, including the loss function and optimizer. Post-processing methods for brain tumor segmentation are also introduced. Finally, we introduce the evaluation criteria for the brain tumor segmentation task, and report the results consisting of the ablation experiment and comparison with the state-of-the-art methods.

### 3.1 Brain Tumor Dataset and Pre-Processing

In this section, we present the details of experimental data, it includes brain tumor datasets, data preprocessing and data augmentation.

#### 3.1.1 Brain Tumor Datasets

The brain image dataset is provided by MICCAI Multimodal Brain Tumor Segmentation Challenge (BraTS) (39, 40). Each sample of the patient includes four modalities. The brain tumor datasets were collected from 19 institutions with the same resolution of 1 mm^3^, and were unified to the same anatomical template. The size of each modality was 240x240x155. All BraTS multimodal datasets include four modals, which are native (T1), post-contrast T1 weighted (T1Gd), T2 weighted (T2), and T2 fluid attenuation inversion recovery (T2-FLAIR). **Table 2** summarizes the dataset of BraTS 2017-2020. The training datasets of BraTS 2018-2020 are used to train our network.

**Table 2:** Summary of the BraTS challenge dataset from 2017 to 2020.

Dataset	Training	Validation	Testing
BraTS 2017	285	46	146
BraTS 2018	285	66	191
BraTS 2019	335	125	166
BraTS 2020	369	125	166

#### 3.1.2 Pre-Processing

Due to different data collection agencies, there are differences in the pixel intensity. In order to make the deep learning network learn more uniform and the segmentation features more accurate, it is necessary to use image pre-processing methods to standardize the data.

In the dataset provided by BraTS 2020, the brain area occupies less than 50% of the total area. A large background area increases the proportion of negative samples, making it difficult for deep learning networks to effectively learn brain tumor features (16). In addition, more tumor pixels are incorrectly classified as background. Different from (41, 42), which crops images into small patches, we crop each image to a size of 144x176 to preserve as much brain region information as possible and reduce the interference of background regions. Specifically, we keep the center area of each image and cropped the edge area. Maximizing the preservation of brain information in non-tumor areas is beneficial for the network to better learn to distinguish the difference between tumor and normal brain tissue. After cropping the image, we use min-max normalization (43) to process the image to reduce the difference between the data collected by different institutions. Specifically, we calculated the maximum and minimum pixel intensity of the 3D brain data of each brain tumor patient in a single modality, and normalized the value range of each pixel to 0 and 1 through min-max normalization between. Performing min-max normalization on a single modality of each sample can not only reduce the difference between scans from various institutions, but also avoid the difference of various scans from the same institution. In addition, normalizing the pixel value between 0 and 1 facilitates the back propagation of gradient during the training process.

#### 3.1.3. Data Augmentation

In order to solve the problem of less training data, we also carried out data augmentation operations. Data augmentation can effectively increase the sample size and prevent the model from overfitting. Commonly used data augmentation methods include flipping (44), transposing, and rotating (45). In order to ensure that the pixel intensity of data does not change significantly and to make the network robust to the shape of tumor, we use the data augmentation strategy of flipping. This strategy can enable the deep learning network to learn the shape characteristics of brain tumors, and use the shape information of brain tumors and non-tumor regions to help the network distinguish tumor regions with similar pixel intensity from normal brain tissue regions.

### 3.2 Loss Function

In the brain tumor images, the proportion of the lesion area is small, in other words, the foreground area is much smaller than the background area. Class imbalance makes it difficult for some commonly used segmentation loss functions to train network parameters effectively. In order to reduce the impact of class imbalance on network training, the network is trained with a combination of dice loss (42) and cross-entropy loss. The joint loss combining dice loss and cross-entropy loss is proven to have an excellent performance in medical image segmentation tasks (46).

Dice loss is a similarity measure method, which is widely used in medical image segmentation, and its value range is [0, 1]. Dice loss can be expressed as follows:


(8) $$L_{dice}=\frac{2\Sigma_{i=1}^{Z}p_{i}q_{i}}{\Sigma_{i=1}^{Z}p_{i}^{2}+\Sigma_{i=1}^{Z}\;q_{1}^{2}},$$

where *Z* denotes the sums of voxels, *p_i_* ∈*P* is the predicted binary segmentation volume, and *q_i_* ∈*Q* is the ground truth of segmentation volume.

Dice loss focuses on the segmentation results of the foreground regions, so it can improve the impact of class imbalance. But when the foreground area in the image is too small, the predicted segmentation result has a greater impact on the calculation result of loss function, making the training unstable. Therefore, we combine dice loss and cross-entropy to improve the training stability. The loss function of brain tumor segmentation network without deep supervision can be expressed as follows:


(9) $$L_{m}(\omega_{m};D)=1-\frac{1}{N}\Sigma_{i=1}^{N}\log\;f_{m}{\left(\omega_{m};x_{i}\right)}^{\left(yi\right)}-\frac{2\Sigma_{i=1}^{N}y_{i}{\hat{y}}_{i}}{\Sigma_{i=1}^{N}y_{i}^{2}+\Sigma_{i=1}^{N}{\hat{y}}_{i}^{2}},$$

where the brain tumor dataset *D* including *N* examples, *x*_i_ is the *i^th^* image of brain MRI scans, and *y_i_* is the ground truth corresponding to *x_i_*. ${\hat{y}}_{i}$ denotes the predicted binary segmentation result corresponding to *x_i_*.

### 3.3 Implementation Details

Our framework was constructed using the TensorFlow2 (47) libraries. The GPU used in the experiment is a virtualized NVIDIA Tesla V100 with only 16 GB of memory. Its computing performance is a quarter of that of a physical GPU. For the training of our method, the total number of epochs is set to 50 and the batch size is set to 32. Adam optimizer (48) is used to optimize the training for all experiments. Adam optimizer, combining the advantages of the AdaGrad and RMSProp optimization algorithms, comprehensively considers the first moment estimation (First Moment Estimation, the mean value of gradient) and the second moment estimation (Second Moment Estimation, the uncentered variance of gradient), and calculate the update step size. The update of parameters of the Adam optimizer is not affected by the scaling transformation of the gradient. It is suitable for the unstable objective function and problems with sparse gradients or very noisy gradients. In our method, the initial learning rate of the Adam optimizer is 1*e* ^–4^, the algorithm of learning rate decay is like as (24).

### 3.4 Post-Processing

In order to further improve the accuracy of the brain tumor segmentation results, we performed post-processing operations on the output of the network. Commonly used post-processing methods for image segmentation include thresholding, erosion, dilation, open operations, close operations, and CRF. For brain tumor segmentation tasks, the pixel intensity and the morphology features of some brain tissues in the brain image are similar to the tumor area, it is easy to interfere with the segmentation of the tumor area, resulting in false positives segmentation results. Through observation, the normal area that is misclassified as a tumor is usually small. In order to reduce the influence of false positives on the segmentation accuracy, we concatenate all the 2D segmentation results of each patient into 3D voxels. And then, we calculate the volume of each independent predicted brain tumor area in each 3D voxel and eliminate the smaller predicted tumor. We keep the largest predicted tumor in each patient and use its volume as the baseline. Then, we compare the volume of other predicted tumors with the baseline. When the volume of other predicted tumors is less than one-tenth of the baseline, we determine that these predicted tumors are false positives.

### 3.5 Evaluation Metrics

In order to evaluate the segmentation performance of brain tumors more comprehensively, dice similarity coefficient (DSC), sensitivity, specificity, and hausdorff distance (HD) are used as evaluation metrics. All evaluation metrics can be expressed as follows:


(10) $$\text{Sensitivity}=\frac{TP}{TP+FN},$$

(11) $$\text{Specificity}=\frac{TN}{TN+FP},$$

(12) $$\text{DSC}=\frac{2\left|U\cap V\right|}{\left|U\right|\cup \left|V\right|},$$

(13) $$\text{Hausdorff}=\text{max}\{\underset{u\in U}{\text{max}}\underset{v\in V}{\text{min}}(u,v),\underset{v\in V}{\text{max}}\underset{u\in U}{\text{min}}(u,v)\},$$

where true positive (*TP*), true negative (*TN*), false positive (*FP*), and false negative (*FN*) are usually used to calculate the evaluation metrics in the segmentation methods. Higher values of sensitivity indicate that the larger tumor area is segmented correctly. Higher values of specificity indicate that the larger non-tumor area is segmented correctly. *U* and *V* indicate the ground truth of the lesion area and the prediction of network, respectively. Higher values of DSC indicate that the segmentation of the lesion area is more accurate. *u* and *v* indicate the set of points on the boundary of ground truth *U* and the set of points on the boundary of prediction *V*, respectively. Lower values of Hausdorff distance indicate that the segmentation of the lesion area is more accurate. In this paper, we use Hausdorff95, which is based on the calculation of the 95^th^ percentile of distances between the boundary points in the ground truth and prediction. Due to the presence of outliers in the boundary area, hausdorff95 can avoid the interference of outliers on the segmentation performance.

### 3.6 Evaluation on Model Architecture

We present a detailed study of the proposed network on the MICCAI Multimodal Brain Tumor Segmentation Challenge 2020 in this section. The training dataset provided by BraTS 2020 is used to train the network. In order to evaluate the segmentation performance of our method more objectively, we upload the predicted results of the validation dataset to the Image Processing Portal (IPP) of CBICA’s.

Similar to the training dataset, the validation dataset also includes four modal brain MRI scans. The validation dataset consists of a total of 125 brain data of patients, and for the axial axis, each brain MRI scans of the patient consisted of 155 images with a size of 240x240. The validation dataset contains mixed glioblastoma (GBM/HGG) and lower grade glioma (LGG). In order to match the trained network input, we use the same cropping method as the training dataset to reduce the image size of each validation dataset to 144x176. After obtaining the prediction results, we restore each image to its original size and submit it to the online evaluation system.

#### 3.6.1 Study of Attention Pre-Activation Residual Module

APR module is modular so that it can be easily added to the segmentation structures. In our proposed model, the APR module is used in the encoder part to improve the ability of extracting tumor features. Three structures are designed to compare with the APR module. The first structure does not use the shortcut and attention gates. The second structure adds the shortcut, but there are no attention gates. The third structure uses the shortcut, and the use of the attention gates is consistent with (31), in other words, combine attention gates with the skip connections.

In **Table 3**, we report the results of the comparative experiment. The results on whole brain tumor predictions demonstrate that the APR module has achieved the first place in three evaluation metrics of dice similarity coefficient, sensitivity, and Hausdorff distance. Due to the large proportion of negative samples, the specificity scores of the four structures are very similar. In addition, the structure of the Attention U-Net has a better segmentation performance for brain tumors, which also proves that the attention gates are helpful for the fusion of multi-scale features. However, for brain tumor segmentation tasks, too large feature scale differences cannot make attention gates accurately weight ROI. This result proves that the APR module contributes to brain tumor segmentation tasks.

**Table 3:** Ablation experiment of the APR module without multi-task deep supervision on the BraTS 2020.

Method	DSC (%)	Sensitivity (%)	Specificity (%)	Hausdroff95
without shortcut & AGs	88.57	88.56	99.89	7.03
without AGs	88.69	88.01	**99.90**	6.99
Attention UNet	88.89	89.25	99.88	6.78
APR module	**88.95**	**89.56**	99.89	**6.51**

The bold values indicate the best results.

#### 3.6.2 Study of Multi-Task Deep Supervision

MTDS is used to extract richer visual features. It can be applied to multi-label segmentation tasks similar to brain tumor segmentation. We design three comparative structures. The first structure does not use deep supervision. The second structure adds deep supervision, but only uses the whole brain tumor mask as the label for all branches. The third structure uses MTDS, and uses enhancing tumor, the necrotic and the non-enhancing tumor, and peritumoral edema as the labels of the three branches, respectively.

**Table 4** shows the comparison experiment results of MTDS and the other two structures. The structure with the MTDS strategy has achieved the top rank in all evaluation metrics. Through the comparative experiments, we can find an interesting phenomenon. The segmentation results of structure without deep supervision are better than the structure with single-task deep supervision in the evaluation metrics of DSC, Sensitivity, and Hausdorff95. Although deep supervision techniques can alleviate the difficulty of optimization arising from gradient flow, it interferes with the hierarchical representation generation process. Due to the inconsistency of optimization objectives, the positive optimization effect on the shared shallow parameters is small, which reduces the accuracy of brain tumor segmentation.

**Table 4:** Ablation experiment of deep supervision on the BraTS 2020.

Method	DSC (%)	Sensitivity (%)	Specificity (%)	Hausdroff95
without deep supervision	88.95	89.56	99.89	6.51
single-task deep supervision	88.74	87.95	99.90	6.84
multi-task deep supervision	**89.18**	**89.24**	**99.91**	**5.77**

The bold values indicate the best results.

### 3.7 Comparison with State-of-the-Art Methods

Our proposed model is evaluated on the public BraTS 2020 validation dataset to compare its performance with the state-of-the-art methods which are on the BraTS2017, BraTS2018, and BraTS2019 leader board. The results of our method comparison with the state-of-the-art methods are reported in **Table 5**.

**Table 5:** The results of comparison between our proposed method and state-of-the-art methods.

Method	Dataset	DSC (%)	Sensitivity (%)	Specificity (%)	Hausdroff95
BCVUniandes (49)	2017	86.8	84.2	99.5	18.456
BRATZZ27 (50)	2017	88.0	85.6	99.6	5.72
CISA (50)	2017	87.3	85.4	99.4	5.18
CMR (50)	2017	85.6	81.1	99.6	5.87
MIC_DKFZ (50)	2017	90.2	90.1	99.5	6.77
Zhouch (50)	2017	**90.3**	90.3	99.5	**4.74**
RadCNN (51)	2017	89.0	89.1	99.5	6.53
Radiomics-miu (52)	2018	87.6	86.2	99.5	4.90
GBMNet (50)	2018	88.3	**93.4**	98.9	5.46
Mmonteiro2 (50)	2018	87.0	87.4	99.3	5.79
UNetImage (50)	2018	89.9	91.0	99.4	5.10
RA-UNet (50)	2018	89.1	89.4	99.3	5.87
Voxel-GAN (53)	2018	84.0	86.0	99.0	6.41
S3D-Unet (41)	2018	88.7	90.1	99.4	5.51
3D Dense U-Nets (54)	2018	88.9	88.0	98.0	7.27
3D Attention UNet (55)	2019	89.8	90.0	99.4	6.29
MECU-Net (56)	2019	90.2	90.8	99.5	5.41
Multi-step cascaded network (57)	2019	88.6	92.1	99.2	6.23
3D U-Net (58)	2019	89.4	89.7	99.5	5.68
Our method	2020	89.2	89.2	**99.9**	5.77

The bold values indicate the best results.

Most state-of-the-art methods ensemble the segmentation results of multiple models, and the segmentation results of ensemble of multiple models is usually better than a single one. In order to show the performance of our proposed method more visual, we did not use the ensemble of multiple models, but only used the proposed single model to compare with other methods. For the whole brain tumor segmentation task, the Dice score of whole tumors reached 0.86-0.90, the Sensitivity score of whole tumors reached 0.85-0.92. Specificity scores of all methods are very high, almost over 0.99. The Hausdorff distance is basically between 4 and 7. The experimental results show that our method has a strong competitiveness.

**Figure 5:**
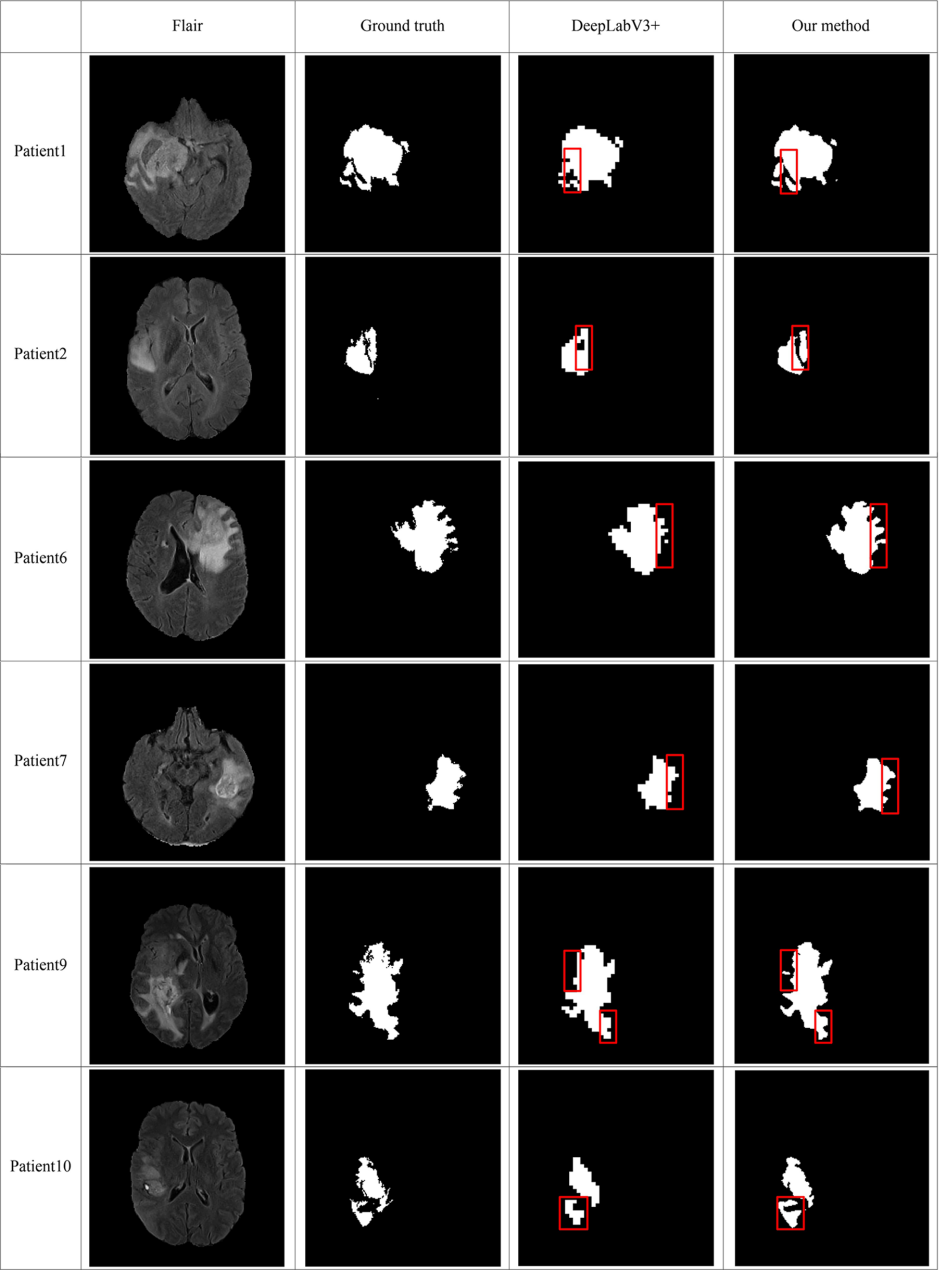
Comparison of brain tumor segmentation results between our method and DeepLabV3+. The differences between the segmentation results of the two methods are marked by the red boxes.

In order to make the comparison result more objective, we retrain several state-of-the-art segmentation models to the brain tumor dataset and evaluated them on the BraTS2020 dataset. It can be seen from **Table 6** that our method has achieved the first place in the DSC, Sensitivity, Specificity, and Hausdorff distance. At the same time, our method has the least number of parameters. **Figure 5** shows a more intuitive comparison between the segmentation results of our method and state-of-the-art methods.

**Table 6:** The results of comparison between our proposed method and state-of-the-art methods on the BraTS 2020.

Method	DSC (%)	Sensitivity (%)	Specificity (%)	Hausdroff95	Parameter
U-Net (10)	87.59	87.04	99.89	8.97	34.5M
ResU-Net (59)	87.06	86.63	99.80	9.16	8.2M
ResU-Net++ (60)	88.48	87.98	99.90	7.42	42.2M
DeepLabV3+ (61)	82.99	84.16	99.82	11.05	39.4M
PSPNet (62)	83.74	82.27	99.87	5.99	35.0M
Attention UNet (31)	87.58	87.26	99.82	8.55	9.3M
Our method	**89.18**	**89.24**	**99.91**	**5.77**	**3.3M**

The bold values indicate the best results.

## 4. Discussion and Conclusion

Brain tumor is a disease that threatens human health. Manual segmentation is time-consuming and subjective. The difficulties of the automatic brain tumor segmentation technology include the sensitivity of the algorithm to tumor regions and the suppression of response to non-tumor regions. In order to improve the ability of the convolutional neural networks to locate ROI, we propose the APR module. This module uses the residual units and attention gates to construct a multi-scale feature fusion method. The simple fusion of low-level feature and the high-level feature of residual unit pass the features of non-interest region to the deeper layers of network. It interferes with the extraction of important information about brain tumors from the encoder part. The attention gate added in the residual unit focus attention on the tumor area, reduced the response of non-interest areas, thereby improving the ability of the convolutional neural network to locate the area of interest. This method has proved its superiority in brain tumor segmentation experiments.

In order to improve the utilization of multi-modal information in brain tumor segmentation tasks, we propose a MTDS method. Different modalities have different sensitivities to the tumor area. In order to fully explore the potential information of multimodal data, we have designed multiple branches in the network, and each branch is used to complete a specific task. In order to avoid the chaotic design from interfering with the ability of the network to extract tumor features, we designed a MTDS method for the characteristics of different tumor regions. In addition, MTDS helps the network to extract richer semantic features and alleviate the problem of network overfitting. We also tested its performance on the brain tumor segmentation task, and the results of experiment proved our hypothesis. The experimental results show that our model has a generalization ability and extension possibilities.

In this paper, we focus on the segmentation accuracy and robustness of a single network to the target region. We hope to design a simple and easy-to-use 2D segmentation method to reduce the dependence of network training on the hardware and reduce training time. Due to the few network parameters, our proposed method is not as good as some segmentation results that integrate multiple 3D networks. In future work, we will continue to focus on the improvement of the current method to make it smaller and more flexible, and at the same time have a higher segmentation accuracy. In order to achieve this goal, we will improve the currently proposed attention mechanism to enable it to integrate richer multi-scale features. In addition, we will make the architecture much more general to other medical image segmentation datasets.

## Data Availability Statement

The original contributions presented in the study are included in the article/supplementary material. Further inquiries can be directed to the corresponding author.

## Author Contributions

SM and FG contributed to the conception of the study. SM performed the experiment. FG contributed significantly to analysis and manuscript preparation. SM performed the data analyses and wrote the manuscript. JT and FG helped perform the analysis with constructive discussions. All authors contributed to the article and approved the submitted version.

## Funding

This work is supported by a grant from National Key R&D Program of China (2020YFA0908400) and National Natural Science Foundation of China (NSFC 61772362, 61972280).

## Conflict of Interest

The authors declare that the research was conducted in the absence of any commercial or financial relationships that could be construed as a potential conflict of interest.

## Publisher’s Note

All claims expressed in this article are solely those of the authors and do not necessarily represent those of their affiliated organizations, or those of the publisher, the editors and the reviewers. Any product that may be evaluated in this article, or claim that may be made by its manufacturer, is not guaranteed or endorsed by the publisher.
